# Development of Sensitive and Reliable UPLC-MS/MS Methods for Food Analysis of Emerging Mycotoxins in China Total Diet Study

**DOI:** 10.3390/toxins11030166

**Published:** 2019-03-17

**Authors:** Danlei Sun, Nannan Qiu, Shuang Zhou, Bing Lyu, Shuo Zhang, Jingguang Li, Yunfeng Zhao, Yongning Wu

**Affiliations:** China National Center for Food Safety Risk Assessment, Key Laboratory of Food Safety Risk Assessment, National Health Commission, Beijing 100021, China; sundl603@gmail.com (D.S.); qiunannan@cfsa.net.cn (N.Q.); lvbing@cfsa.net.cn (B.L.); zhangsh@cfsa.net.cn (S.Z.); lijg@cfsa.net.cn (J.L.); zhaoyf@cfsa.net.cn (Y.Z.)

**Keywords:** emerging mycotoxins, complex food matrices, UPLC-MS/MS, total diet study

## Abstract

With the climatic changes that have taken place during the last decade, the spectrum of fungal pathogens as well as mycotoxins has considerably changed. As a result, some emerging mycotoxins have been shown to occur frequently in agricultural products. In this study, a sensitive and reliable method for the determination of 10 emerging mycotoxins (beauvericin, enniatin A, enniatin A1, enniatin B, enniatin B1, alternariol, alternariol monomethyl ether, altenuene, tentoxin, and tenuazonic acid) in 12 different food matrices (cereals, legumes, potatoes, meats, eggs, aquatic foods, dairy products, vegetables, fruits, sugars, beverages, and alcohol beverages) was developed and validated. After a simple extraction, a one-step sample clean-up by a HLB solid phase extraction (SPE) column was sufficient for all 12 food matrices prior to analysis with ultra-high performance liquid chromatography coupled to tandem mass spectrometry (UPLC-MS/MS). Isotope internal standards ^13^C-TeA, TEN-d_3_, and ^13^C-AFB2 were used for accurate quantification. Validation in terms of linearity, selectivity, sensitivity, accuracy, and precision (intra and inter-day variability) were evaluated for the 10 mycotoxins in all selected matrices. The sensitivity varied from 0.0004 to 0.3 ng mL^−1^ (limits of detection) and from 0.002 to 0.9 ng mL^−1^ (limits of quantitation). The recoveries of 10 mycotoxins in fortified samples were from 60.6% to 164% including very low spiking levels in all 12 food matrices, with relative standard deviations (RSDs) less than 12%. The proposed methodology was applied to the analysis of 60 samples collected from five provinces within the 6^th^ China Total Diet Study with the results discussed in detail. The advantages of sensitivity, accuracy, and robustness made it a powerful tool for emerging mycotoxin monitoring and dietary exposure assessment.

## 1. Introduction

Fungi of the genus Alternaria are ubiquitous saprophytes distributed in soil, household dirt, and decaying organic material and can cause the spoilage of fruits and vegetables during transport and storage [1]. It commonly occurs worldwide and infects various cereal crops, oilseeds, fruits, and vegetables such as wheat, sorghum, barley, apples, citrus fruits, tomato, and carrots [2,3,4,5,6,7,8,9]. Alternaria species produce several toxic secondary metabolites called alternaria mycotoxins (ATs), which include tenuazonic acid (TeA), alternariol (AOH), altenuene (ALT), alternariol monomethyl ether (AME), and tentoxin (TEN) [2]. Tenuazonic acid (TeA) has acute toxic effects on various mammals reviewed by Ostry [10] and Gruber-Dorninger et al. [11]. Although most alternaria toxins exhibit only low acute toxicity, there is strong evidence that AOH and AME are mutagenic, cytotoxic, genotoxic, and play an important role in the etiology of human esophageal cancer [12,13,14,15]. In this regard, the European Food Safety Authority (EFSA) set the tolerable daily intakes (TDI) at 2.5 ng/kg body weight per day for AOH and AME and 1500 ng/kg body weight per day for TeA and TEN [16,17]. Based on these thresholds, the maximum levels allowed for these toxins in food might be released by some organization and regulated in the future.

Enniatins (ENNs) and beauvericin (BEA) are a group of cyclic hexadepsipeptides consisting of alternating hydroxy acid and N-methylamino acid residues that have been discovered as early as 1947 in a strain of Fusarium oxysporum, known as emerging Fusarium mycotoxins [18]. BEA has phenyl substituents on the N-methylamino acid residue whereas ENNs have various aliphatic substituents at the same positions. They are secondary fungal metabolites produced by several Fusarium such as Beauveria, Paecilomyces, Polyporusm, and Verticillium, which are parasitic to maize, wheat, rice, and other cereals [19,20,21,22]. High levels of BEA in wheat samples reaching 4 mg/kg were reported in the Mediterranean area in a recent study [23]. BEA and ENNs share similar chemical structures and thus have similar toxic mechanisms of action including interference with the electrochemical gradient of the cell membranes by disturbing the normal physiological level of cations [24,25]. BEA can also induce apoptosis and DNA fragmentation [26], and has been proved to have genotoxicity [27,28].

A variety of the methods used in the analysis of emerging mycotoxins such as TLC, HPLC-UV, and HPLC-FLD are vulnerable to interference from co-eluting compounds and have difficulties in dealing with multiple analytes, especially if complicated food matrices are analyzed [29,30,31,32,33]. Recently, LC-MS/MS was used for the accurate determination of emerging mycotoxins in food matrices and has increasingly become the preferred technique due to its high selectivity and sensitivity for multi-mycotoxins analysis [34,35,36,37,38,39,40,41,42]. These methods started by targeting only a few toxins in limited food categories such as enniatins and beauvericin in maize [34] and eggs [35], AOH and AME in cereal, fruit, and vegetable products [38], and six Alternaria toxins in tomatoes [39]. Then, modified forms (mainly glucosides and sulfates) were taken into consideration and more food matrices were investigated [40,41,42]. The complexity of food matrices presented a big challenge to the sample preparation, HPLC separation, and matrix effect of mass spectrometry, especially when lacking the corresponding isotope-labeled internal standards. Usually, a solid phase extraction or QuEChERS purification is required to reach satisfying sensitivity and accuracy. Until now, the most frequently tested foods have been cereals, tomato-based products, eggs, fruit juices, wine, maize, and sunflower seeds [43].

Total diet study (TDS) is considered as the most efficient and effective method to provide reliable estimates of the dietary intakes of certain chemical substances for general populations recommended by the World Health Organization (WHO) [44]. China has successfully conducted five total diet studies. The strategy of the 6^th^ China TDS followed a similar procedure as the previous ones including dietary survey, food aggregation, collection of individual samples, cooking and preparation of food composite samples, experimental analysis of food samples, and dietary exposure assessment. The geographical areas involved in this new round of the TDS were further enlarged from 20 to 24 provinces (municipalities, autonomous regions) that represented the dietary habits in various regions of China and covered more than two-thirds of the total Chinese population. The per capita consumption of foods was classified into 13 categories according to the food composite approach adopted in this study including cereals and cereal products, legumes and related products, potatoes and potato products, meats and meat products, eggs and egg products, aquatic foods and aquatic foods products, milk and dairy products, vegetables and vegetable products, fruits and fruit products, sugar and sugar products, alcohol beverages, and condiments (including cooking oils) that were put into the other 12 categories during cooking. Mycotoxins have been included in several previous national or regional TDSs such as the first and second French TDS [45], Spanish TDS [46], Lebanese TDS [47], Netherlands TDS [48], Hong Kong TDS, and the fourth [49] and fifth [50] China TDS (38 mycotoxins); however, none of them included emerging mycotoxins.

This study aimed to develop a method to simultaneously quantify 10 emerging mycotoxins (TeA, ALT, AME, AOH, TEN, BEA, ENNA, ENNA1, ENNB, and ENNB1) in 12 different food matrices with good accuracy and precision that was applicable for a total diet study. A sensitive and reliable UPLC-MS/MS method following an optimized solid-phase extraction (SPE) procedure was successfully developed. Ten emerging mycotoxins were completely separated under a 12-min chromatographic gradient elution. After validation, the proposed method was used to analyze 60 samples collected from five provinces within the 6^th^ China TDS, demonstrating the reliability and broad applicability of the proposed method. The advantages of sensitivity, accuracy, and robustness made it a powerful tool for emerging mycotoxin monitoring and dietary exposure assessment.

## 2. Results and Discussion

### 2.1. Optimization of Tandem Mass Parameters

The MS/MS conditions were optimized on a Triple Quad 6500+ mass spectrometer by the individual infusion of each compound. To obtain the most intense signals of the precursor ions, the key parameters that influence the ionization procedure such as ionization mode, ion spray voltage, declustering potential, curtain gas, source temperature, ion source gas 1 (sheath gas), and ion source gas 2 (drying gas) were manually optimized in steps. For AME and TeA, the negative mode with a spray voltage of −4.5 kV generated the highest precursor ions, whereas the positive mode with a spray voltage of 5.5 kV was best for the other eight analytes. On this basis, the collision energy (CE) for each compound was tuned up individually to produce the most sensitive and stable product ions. Multi reaction monitoring (MRM) mode was used in mass detection including soft ionization, trapping precursor ions, fragmentation to product ions, and quantification. In this mode, transitions between a precursor ion and the two most abundant fragment ions were chosen for each analyte. More detailed information of the MRM parameters of the analytes are summarized in Table 1. The peak areas of quantification ions were used to estimate the concentration. The abundance of the confirmation ions relative to that of the quantification ion was used as the identification criteria. The ratio should meet the requirement of Commission Decision 2002/657/EC.

### 2.2. Chromatographic Separation

**Figure 1:**
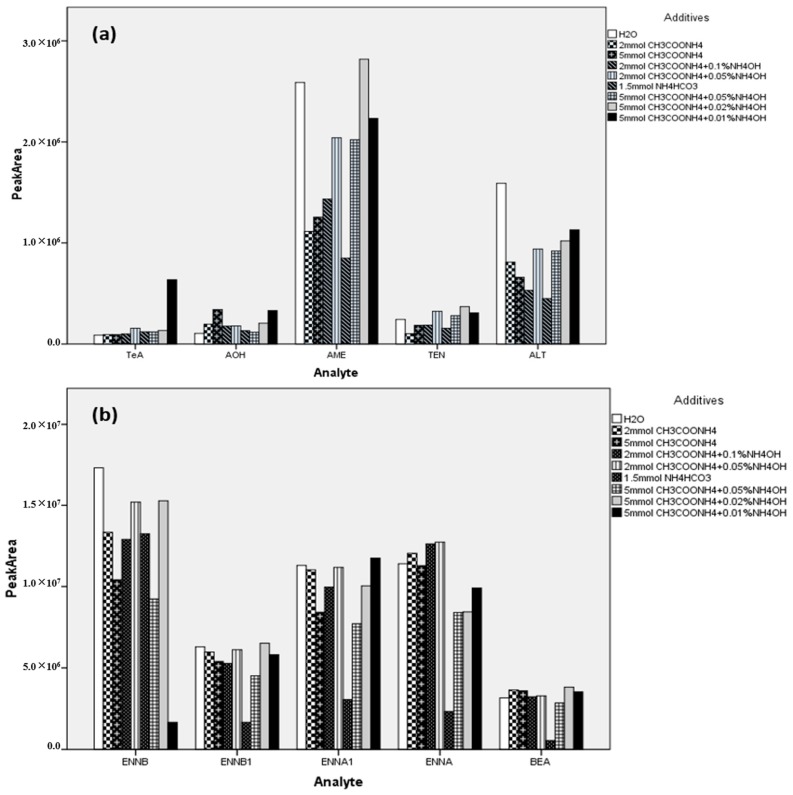
Effects of additives in the mobile phase on the mass signal intensities for ATs (**a**), ENNs and BEA (**b**). Abbreviations: NH_4_HCO_3_, ammonium bicarbonate; CH_3_COONH_4_, ammonium acetate; NH_4_OH, ammonia water solution.

**Figure 2:**
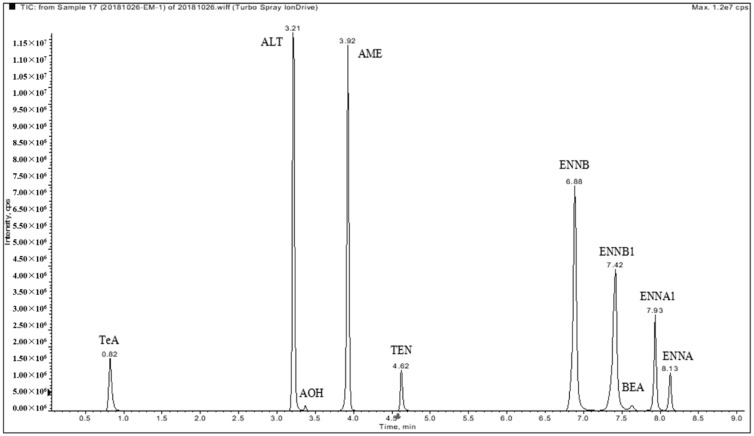
An overlapped chromatogram of a standard mixture of 10 emerging mycotoxins (100 ng/mL of each) plotted by the extraction of their individual quantification ions, showing a complete UPLC separation.

The baseline-separation of the 10 target compounds have not been previously reported. The key parameters that affect chromatographic behavior were carefully investigated including column, mobile phase (organic modifier), additives (ammonium acetate, ammonium formate, ammonium bicarbonate, and ammonium hydroxide) at different concentrations, flow rate, gradient program, and column temperature. The separation was achieved on a CORTECS C18 UPLC column, which enabled the satisfactory resolution and sharp peaks for all of the analytes. An organic modifier (acetonitrile or methanol) in the mobile phase has a very slight influence on the chromatographic separation. Due to the clean background signal, acetonitrile was preferred as the organic modifier. Ammonium acetate, ammonium formate, and ammonium hydroxide at different concentrations were evaluated as additives and did not contribute to the intensity of ENNs and BEA, but significantly affected the ion signals of ATs (see Figure 1). It can be observed that the aqueous mobile phase containing ammonium acetate and ammonia gave rise to much better signals for all of the ATs. Particularly for TeA, 5 mmol/L ammonium acetate and 0.01% ammonia markedly enhanced the intensity more than three times higher than that of the other conditions. As a result, water with 5 mmol/L ammonium acetate and 0.01% ammonia (solvent A) and acetonitrile (solvent B) was chosen as the mobile phase for further optimization of the gradient program to achieve the satisfactory separation of all analytes. A representative chromatogram of the mixed standards of 10 emerging mycotoxins at 100 ng mL^−1^ is presented in Figure 2.

### 2.3. Sample Preparation

The extraction and clean-up procedure is the most important step in multi-mycotoxin methods, especially when various different types of complex food matrices need to be analyzed for a total diet study. In previous works, several solvents have been successfully used for the extraction of emerging mycotoxins from certain food matrices [51,52,53,54,55,56]. Due to the weak acidity of TeA and AOH, a phosphate buffer (0.05 mol L^−1^, pH 3.0) was used to prepare the extraction solvent to enhance the solubility of acid compounds in the acetonitrile/methanol/water (45/10/45) mixture.

**Figure 3:**
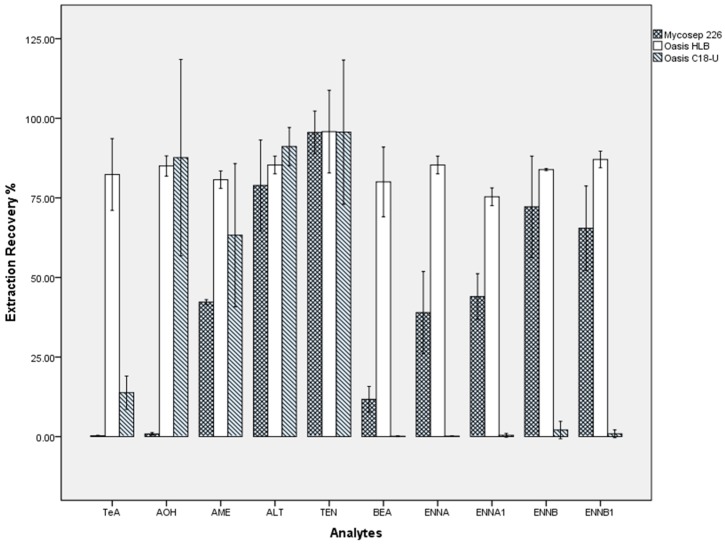
Recovery using two SPE cartridges and a Mycosep 226 purification cartridge for 10 emerging mycotoxins (ALT, AOH, AME, TEN, TeA, BEA, ENNA, ENNA1, ENNBm and ENNB1) spiked at 100 ng mL^−1^.

Total diet samples contain diverse ingredients (fat, proteins, nutrients, carbohydrate, etc.) that may markedly influence the ionization of target compounds or increase the risk of clogging. Accordingly, a purification step that can remove most of the matrix interference and obtain a satisfying recovery of the target analytes is required. Several cartridges used in our previous works for mycotoxin extraction were evaluated including Mycosep 226, Oasis HLB, and Oasis C18 cartridges. The Mycosep 226 multifunctional cartridge is a one-step clean-up column that absorbs and removes the impurities while allowing the target analytes to pass through. This was successfully applied to the measurement of 33 mycotoxins in food samples from the 5^th^ China TDS [50]. Two types of conventional reverse-phase SPE cartridges, Oasis HLB and C18, are also powerful tools that have been widely used in mycotoxin analysis. Herein, the standard solution of 10 emerging mycotoxins was treated by the three cartridges to assess their recovery performance. According to the difference in adsorption theory and the manuals provided by the supplier, the three cartridges were operated following different protocols. For the HLB and C18 cartridges, a standard solution prepared in acetonitrile/water (10/90) was loaded, and 5 mL methanol/water solution (V/V = 20/80) was used to wash the cartridge followed by elution with 5 mL methanol and then 5 mL acetonitrile. The eluent was nitrogen-dried and reconstituted prior to UPLC-MS/MS analysis. For the Mycosep 226 cartridge, a standard solution prepared in acetonitrile/water (86/14) was allowed to pass through and was collected for the nitrogen-dried, reconstituted, and UPLC-MS/MS analysis. Figure 3 demonstrates the percentage recoveries obtained from the Mycosep 226, HLB, and C18 cartridges for the 10 emerging mycotoxins. The C18 cartridge presented high recoveries only for AOH, AME, ALT, and TEN. This could be because the polar compound TeA may have low adsorption on the C18 sorbent, while the ENNs and BEA might not be effectively eluted. For the Mycosep 266 cartridge, the recoveries of the 10 mycotoxins largely varied from 0% to nearly 100%, which was attributed to the retention on the sorbent and loss in the collected portion. The best recovery performance was obtained on the Oasis HLB cartridge, achieving optimal extraction recoveries of 76%~103% for all 10 emerging mycotoxins. Therefore, this cartridge was used in further optimization to be applicable to the 12 categories of food matrices. The final protocol was determined as stated in the Methods Section. It is noteworthy that filtration through a 0.22 μm filter membrane is commonly needed to remove the insoluble substances and particles prior to sample injection. However, AME, AOH, and ENNs can partially bind to several types of filter membrane, so centrifuging the reconstitution solution at 20,000 rpm for 30 min before UPLC-MS/MS analysis was recommended.

### 2.4. Method Validation

Method validation was carried out according to the guidelines established by the EC [57] and EMEA [58] in terms of the determination of linearity, selectivity, limits of detection (LODs), limits of quantification (LOQs), accuracy (recoveries), and precision (intra and inter-day variability).

The linearity was assessed from a calibration curve ranging from LOQ~500.0 ng mL^−1^ for ATs and LOQ~50.0 ng mL^−1^ for the ENNs and BEA on three consecutive days by using linear regression with a 1/x weighting. All mycotoxins exhibited good linearity over the working range with the regression coefficient (R) ranging from 0.989 to 0.998 and the deviations within 15% of the nominal concentrations, indicating a good linear fit for all 10 analytes.

Selectivity represented the ability of a method to differentiate the target analytes and endogenous components in the matrices. This was proved by comparing blank matrices with spiked matrices at very low concentrations for all 12 food categories. No interference signal from the endogenous matrix components at the retention time of each analyte was observed, which demonstrated the high selectivity of the method.

Sensitivities (LOD/LOQ) of the assay were obtained by analyzing spiked food matrices at low levels. LOD was defined at a signal to noise (S/N) ratio of S/N = 3 and LOQ at S/N = 10. For LOQ, both the accuracy and precision should also be within 20%. The sensitivities of each analyte were varied between the 12 food categories, which were better in a simple matrix such as cereal and beverages and lower in a matrix containing complex ingredients such as meat and meat products. AME, TEN, and BEA had similar LOQs ranging from 0.01 μg kg^−1^ to 0.2 μg kg^−1^. The LOQs for TeA, AOH, and ALT were slightly higher between 0.1–1 μg kg^−1^. The ENNs showed the best sensitivities with LOQs in the range of 0.001–0.05 μg kg^−1^. The detailed information is listed in Table 2 and Table S1, demonstrating a good sensitivity when compared to the previous methods as summarized in Table 3.

The apparent recovery (R_A_), extraction recovery (R_E_), and matrix effects (ME) were also evaluated to separately evaluate the sample preparation step and instrumental detection step using the following formulas [66]:

R_E_(%) = C/B × 100%, (1)

ME(%) = B/A × 100%,(2)

R_A_(%) = C/A × 100%, (3)

where A is the average peak area of the analyte in standard solution; B is the average peak area of the analyte in sample matrix spiked after sample preparation; and C is the average peak area of the analyte in the sample matrix spiked before sample preparation. The R_E_ ranged between 51.3%~120.7% and matrix effects between 65.6% and 196.7% were obtained for all of the analytes in the 12 food categories (Table 2). The results demonstrated an effective analyte extraction, while on the other hand, stretched the need of IS compensation.

The accuracy, expressed as the method recoveries (R_M_), as well as the inter-day and intra-day precision were investigated at low, medium, and high spiking levels (2, 20, and 200 μg kg^−1^ for ATs and 0.2, 2, 20 μg kg^−1^ for ENNs and BEA) in a blank food matrix on three different days in six replicates with internal standards correction. Nearly all of the emerging toxins showed satisfying results in 12 food categories with recoveries between 70~130% and intra-day/inter-day precisions lower than 12%, except for certain ENNs at a very low concentration of 0.2 μg kg^−1^ in cereals, potatoes, and eggs, giving slightly higher recoveries reaching 164% (Table 4). This can be partially attributed to the lack of a corresponding isotope labeled standard for the ENNs group. So, ^13^C-AFB1 with a similar polarity and sample preparation recovery was selected as the reference IS for the ENNs quantification.

The TDS composite sample of each food category was made by blending the single samples after cooking with weights proportional to its consumption, which resulted in a more complicated matrix and a dilution of the target analytes. Therefore, high sensitivity and wide applicability are required for the analytical method used in the TDS. The validation parameters achieved in this section demonstrated a qualified method that could guarantee a reliable dietary exposure study and thereafter a risk assessment for emerging mycotoxins.

### 2.5. Application of the Method in the 6^th^ China TDS

The developed and validated method was used to identify the occurrence of 10 emerging mycotoxins in 60 food samples (five per each food category) within the 6^th^ China TDS. Due to the high sensitivity of the method, 75% of the 60 samples were positive for at least one emerging mycotoxin ranging from ppt to low ppb levels (Table 5). Additionally, all of the 12 food categories had detectable emerging mycotoxins, among which six categories (cereals and cereal products, legume and related products, potatoes and potato products, eggs and egg products, vegetables and vegetable products, and alcohol beverages) had a positive rate of 100% (detected in five out of five samples). Sugar and sugar products as well as beverages and water were rarely contaminated with these mycotoxins.

The most frequently found mycotoxins were TeA (45%), ENNB (43.3%), AME (36.7%), and BEA (33.3%) followed by TEN (28.3%), ENNB1 (28.3%), ALT (26.7%), ENNA (21.7%), and ENNA1 (13.3%), while AOH was not detected in any of the samples. For alternaria mycotoxins, the concentrations ranged from <LOD to a maximum value of 27.73 μg kg^−1^ for TeA, 27.53 μg kg^−1^ for TEN, 10.92 μg kg^−1^ for AME, and 8.77 μg kg^−1^ for ALT, much higher than the concentrations of ENNs, which ranged from <LOD to 1.55 μg kg^−1^. It is also worth noting that the highest levels of TeA, TEN, BEA, ENNA, ENNA1, and ENNB1 were found in eggs and egg products, while cereals and cereal products contained the highest concentration of AME and ENNB. Therefore, eggs and cereals should be given more attention in future studies on emerging mycotoxin contamination and risk assessment.

## 3. Conclusions

In this study, a sensitive and reliable UPLC-MS/MS method was developed to determine 10 emerging mycotoxins in 12 different food categories for the total diet study. The LODs and LOQs ranged from 0.0004 to 0.3 ng mL^−1^ and from 0.002 to 0.9 ng mL^−1^, respectively. ENNs exhibited the best sensitivity. Most mycotoxins showed satisfactory accuracy ranging between 70%~130% in 12 food matrices, with intra- and inter-precision within 12%. After validation, the well-established method was successfully employed to study the occurrence of 10 mycotoxins in 60 food samples collected from five provinces within the 6^th^ China Total Diet Study, demonstrating its ultra-sensitivity and wide applicability. The preliminary results showed that except for AOH, all of the mycotoxins were detected in 13.3%~45% of the samples at ppt to low ppb levels mainly in eggs, cereals, legumes, potatoes, and the related products. These food categories need to be given more attention in the dietary exposure assessment of emerging mycotoxins.

## 4. Materials and Methods

### 4.1. Materials and Reagents

Certificated standard solutions of AOH, AME, TeA, BEA, TEN, ALT, and isotope labeled internal standards TEN-d_3_, ^13^C_17_-TeA, and ^13^C_17_-AFB2 were from Romer Labs (Tulln, Austria). ENNs (ENNA1, ENNA, ENNB1, and ENNB) were purchased from PriboLab (Biopolis, Singapore) and stored at −40 °C in the dark. Acetonitrile and methanol were of LC-MS grade; formic acid, ammonium formate, aqueous solution of ammonium hydroxide (25%), ammonium hydrogen carbonate, and ammonium dihydrogen phosphate were of HPLC grade (Fisher Scientific, Leicestershire, UK). The other chemicals or reagents were of analytical grade or better. A mixed standard solution consisting of 5 μg/mL of ATs and 0.5 μg/mL of ENNs and BEA, and a mixed internal standard solution containing 25 ng/mL of ^13^C_17_-AFB2, 0.5 μg/mL of ^13^C_17_-TeA, and 0.5 μg/mL of TEN-d_3_ were prepared in acetonitrile and stored at −40 °C in the dark. These stock solutions were then diluted with the original mobile phase to obtain the appropriate working solutions and wait for the UPLC-MS/MS analysis. The solvent for the sample extraction was prepared by mixing 450 mL acetonitrile, 100 mL methanol, and 450 mL of 0.05 mol L^−1^ phosphate buffer (ammonium dihydrogen phosphate, pH 3.0). The Oasis HLB SPE columns (6 mL with 200 mg stationary phase) were obtained from Waters (Milford, MA, USA).

### 4.2. Food Consumption and Sampling

Food consumption survey was conducted in 24 provinces (municipalities, autonomous region). Six survey sites (two urban sites and four rural sites) were required for provinces with a population of more than 50 million, while there were three survey sites (one urban site and two rural sites) in other provinces. At each site, 30 households were randomly selected to finish both a household survey by a weighing plus 3-day accounting method and an individual survey by a 3-day 24-hour dietary recall method. Food aggregation and consumption of each food item were obtained from the survey.

On this basis, food samples were collected from local food markets such as vegetable markets, grain shops, farmer’s markets, or rural households in each sampling site, and then prepared according to the recipes following the local culinary habits. The composite of each food category was made by blending the prepared food with weights proportional to the average consumption in each province. These provincial composite samples were properly labeled and stored at −20 °C for analysis.

### 4.3. Preparation of Standard Serials and Quality Control Samples

The calibration standard solutions at levels of 0.05, 0.1, 0.2, 0.5, 1, 2, 5, 10, 20, 50, 100, 200, and 500 ng/mL for ATs and 0.005, 0.01, 0.02, 0.05, 0.1, 0.2, 0.5, 1, 2, 5, 10, 20, and 50 ng/mL for ENNs and BEA were made by serial dilutions of the mixed stock solution with acetonitrile/water (10/90, *v*/*v*). Each solution contained 10 ng/mL ^13^C-TeA, 10 ng/mL TEN-d_3_, and 0.5 ng/mL ^13^C-AFB2.

To ensure the accuracy of the analysis, three test samples from each batch of 20 samples were randomly selected and spiked with known amounts of the target analytes, serving as the quality control (QC) samples. The recoveries of the QC samples should be between 60–140% according to the EC guidelines [67].

### 4.4. Sample Preparation

After spiking with the isotope internal standards (^13^C-TeA, TEN-d_3_ and ^13^C-AFB2), 2 g of food sample was incubated with 9 mL of extraction solution for 0.5 h at room temperature, ultrasonized for 0.5 h, and centrifuged at 9000 rpm for 10 min. Exactly 5 mL of the supernatant was mixed with 15 mL 0.05 mol L^−1^ phosphate buffer (ammonium dihydrogen phosphate, pH 3.0). After vortex-mixing for 30 s, the resulting mixture was loaded onto an Oasis HLB SPE column and allowed to pass through the sorbent slowly. The column was sequentially washed with 5 mL of methanol/water solution (V/V = 20/80) and eluted with 5 mL methanol and then 5 mL acetonitrile. A total of 10 mL of the eluent was nitrogen-dried at 40 °C, reconstituted in 1 mL acetonitrile/water (V/V = 10/90), vortexed for 30 s, and centrifuged at 20,000 rpm for 30 min to collect the supernatant prior to LC-MS/MS analysis.

### 4.5. LC-MS/MS Analysis

Analysis was carried out on an Exion LC AD™ System (AB SCIEX, Concord, Ontario, Canada) coupled to a Triple Quad 6500+ mass spectrometer (AB SCIEX). The instrument operation was performed on Analyst^®^ software and the data processing was analyzed on MultiQuant™ software.

#### 4.5.1. Chromatographic Condition

Chromatographic separation of 10 emerging mycotoxins was performed on a UPLC column (CORTECS™ C18, 2.1 × 100 mm, 1.6 μm) under a gradient elution. Acetonitrile (solvent A) combined with aqueous solution containing 5 mmol L^−1^ ammonia acetate and 0.01% ammonium hydroxide (solvent B) served as the mobile phase. After an initial time of 1 min at 8% A, the proportion of A was increased linearly to 35% from 1–4 min, and then increased to 74% within 2 min, held at 74% for 1.5 min, and then increased to 100% within 0.5 min and held for 3 min, finally reduced to 8% within 0.1 min and held for 4 min, with the total runtime of 15 min. The flow rate was set at 0.4 mL/min. The column was maintained at a constant temperature of 50 °C while the temperature of the autosampler was kept at 10 °C.

#### 4.5.2. Mass Spectrometry Condition

The detection and analysis were performed on a Triple Quad 6500+ mass spectrometer, equipped with an ESI source. The instrument parameters in MRM mode in both positive and negative ionization were optimized for each analyte by scanning two fragmentation pathways with the parameters including cone voltage (CV) and collision energies (CE) given in Table 1. Other settings were as follows: curtain gas, 20 psi; source temperature, 450 °C; ion spray voltage, −4500 V and +5500 V, respectively; ion source gas 1 (sheath gas) 60 psi; ion source gas 2 (drying gas) 55 psi; collision gas (nitrogen) medium.

### 4.6. Method Validation

Validation of the method was evaluated referring to the guidelines defined by the European Communities (EC) [57] and European Medicines Agency (EMEA) [58] concerning linearity, accuracy, precision, and sensitivity. Accuracy was investigated from the method recovery (R_M_) at three spiking levels in 12 types of food matrices with internal standard correction.

## Figures and Tables

**Table 1:** MRM transitions of the analytes.

Analyte	Precursor	Quantification Ion	DP/CE ^1^	Confirmation Ion	DP/CE ^1^	Ion Ratio
AOH	258.8	185.1	150/43	213.0	150/37	0.88
AME	270.9	256.0	−110/−29	228.0	−110/−39	0.30
TeA	196.2	139.0	−50/−28	112.2	−50/−34	0.71
TEN	415.3	312.2	120/29	301.9	120/19	0.23
ALT	292.9	275.1	30/13	257.0	30/25	0.70
BEA	784.5	244.2	220/38	262.3	220/34	0.69
ENNA1	668.2	210.0	200/32	228.2	200/33	0.45
ENNA	682.3	210.0	220/34	228.2	220/37	0.41
ENNB1	654.4	196.0	180/33	214.1	180/35	0.76
ENNB	640.3	196.4	180/34	214.2	180/33	0.62
^13^C-TeA	198.2	141.0	−50/−28	114.0	−50/−36	0.50
d_2_-TEN	440.2	404.4	140/35	412.4	140/37	0.31
^13^C-AFB2	332.0	303.2	100/38	273.1	100/45	0.71

^1^ DP, declustering potential (V); CE, collision energy (eV).

**Table 2:** Sensitivity, extraction recovery, and matrix effect of the method for the 12 food categories.

Analyte	R_E_^1^ (%)	Matrix Effect (%)	R_A_^2^ (%)	LOQ (μg kg^−1^)	LOD (μg kg^−1^)
AME	62.4~78.2	71.4~134	55.8~100.8	0.01~0.08	0.003~0.03
TeA	75.6~100.8	65.6~122	61.6~98.6	0.1~0.9	0.04~0.3
TEN	78.2~99.5	99.7~162.7	94.2~140.9	0.05~0.2	0.02~0.05
ALT	73.2~120.7	98.5~190.7	77.3~158	0.2~0.9	0.04~0.3
AOH	53.7~107.5	73.9~143.5	51.5~111.3	0.4~0.9	0.1~0.3
BEA	51.3~113.3	74.5~174.8	53.3~152.3	0.01~0.08	0.002~0.02
ENNA1	55.5~110.7	71.4~188.2	63.8~146.6	0.007~0.06	0.002~0.1
ENNA	54.4~117.0	72.3~155.66	48.4~182.1	0.007~0.06	0.002~0.02
ENNB1	56~89.0	114.4~196.7	76.5~139	0.007~0.06	0.002~0.02
ENNB	54~110.7	115.7~170.0	74.4~135.1	0.002~0.04	0.0004~0.01

^1^ R_E_, extraction recovery; ^2^ R_A_, apparent recovery.

**Table 3:** Analytical methods for emerging mycotoxins in food matrices and their processed products.

Analyte	Matrices	Sample Preparation	Analysis Method	LOQ	References
ATs	Fruit and fruit juices	SPE	UPLC-MS/MS	0.6~3 μg L^−1^	[59]
ATs	Juices, beers, and tomato sauces	SPE	LC-APCI-MS	0.16~12.31 μg kg^−1^	[36]
ATs	Tomato paste	SPE	HPLC-PDA	1.93 μg kg^−1^	[60]
AOH, AME	Tangerines	SPE	HPLC-MS/MS	0.13 μg kg^−1^	[61]
ALT, AOH, AME, ENNB, BEA	Maize and Wheat	Extraction solvent (ACN/water/acetic acid, 79:20:1 *v*/*v*/*v*)	HPLC-MS/MS	0.5~1 μg kg^−1^	[62]
ENNs, BEA	Cereals (wheat, barley, maize, and sorghum)	Extracted by methanol	LC-DAD	400~600 μg kg^−1^	[60]
ENNs	wheat flour and corn grits	SPE	LC-MS/MS	2~3 μg kg^−1^	[37]
ENNs	Rice	Extraction solvent (ACN/water/glacial acetic acid, 79:20:1 *v*/*v*/*v*)	HPLC-MS/MS	0.06 μg kg^−1^	[63]
ENNs	Bread, fruits, vegetables, cheeses, nuts, and jam	Extraction solvent (ACN/water/acetic acid, 79:20:1 *v*/*v*/*v*)	HPLC-MS/MS	0.3~0.9 μg kg^−1^	[64]
BEA and ENNs	Egg	QuEChERS	UPLC-MS/MS	2~10 μg kg^−1^	[55]
BEA and ENNs	Cereals (wheat, Barley, maize, malt and oat)	Bond Elute cartridge	LC-MS/MS	0.9~4.2 μg kg^−1^	[65]

**Table 4:** Accuracy and precision of the method for the 12 food categories (mean and range).

Analyte	Spiked Level (ng/mL)	Measured Value (ng/mL)	R_M_^1^ (%)	RSD (%)	
				Intra-Day	Inter-Day
AME	2	1.68 (1.26~2.63)	84.04 (62.8~131.1)	1.2~11.8	3.6~12.2
	20	16.08 (14.36~21.86)	80.38 (71.8~109.3)	1.2~5.2	3.6~8.7
	200	158.93 (131.00~188)	79.46 (65.5~94)	2.1~7.6	2.8~10.3
TeA	2	2.07 (1.62~2.58)	103.28 (81~129.1)	1.2~6.9	3.3~9.3
	20	18.63 (17.07~20.25)	93.18 (85.3~101.3)	1.4~8.2	3.2~10.9
	200	179.08 (171.4~194)	89.53 (85.7~97)	0.9~7.8	3.2~10.5
TEN	2	2.27 (1.87~2.52)	113.58 (93.5~125.9)	2.2~9.5	4.4~11.8
	20	21.60 (16.35~26.34)	107.11 (81.8~131.7)	1.2~9.2	3.2~10.8
	200	203.21 (167.3~232.9)	99.38 (75.8~116.4)	1.6~6.3	2.3~9.8
ALT	2	2.28 (1.71~2.69)	113.92 (85.5~134.6)	1.6~8.1	3.6~10.5
	20	21.96 (15.98~25.42)	109.79 (80~127.1)	1.2~8.4	6.3~11.3
	200	205.13 (151.5~257.2)	102.58 (75.8~128.6)	1.6~7.8	4.3~10.2
AOH	2	2.06 (1.41~2.88)	102.98 (70.7~143.8)	2.1~8.2	4.8~11.2
	20	17.19 (13.59~24.16)	85.98 (67.9~120.8)	2.4~11.1	5.1~12.3
	200	176.87 (136.2~257.6)	88.43 (68.1~128.8)	1.5~9.8	3.2~10.2
BEA	0.2	0.22 (0.13~0.3)	110.79 (63.1~149.1)	1.5~9.6	4.8~11.2
	2	1.90 (1.31~2.84)	94.89 (65.3~141.9)	2.0~7.7	4.1~9.6
	20	16.77 (12.31~24.75)	83.88 (61.5~123.8)	2.1~9.1	5.3~10.9
ENNA1	0.2	0.23 (0.13~0.31)	114.33 (64.5~154.3)	1.2~8.1	2.4~10.2
	2	1.78 (1.21~2.57)	88.83 (60.6~128.5)	1.3~5.6	2.5~8.8
	20	18.12 (12.22~26.81)	90.62 (61.1~134.1)	2.3~9.2	4.4~11.5
ENNA	0.2	0.22 (0.12~0.33)	107.68 (60.3~164.4)	2.6~6.5	4.8~10.1
	2	1.92 (1.23~2.7)	98.38 (61.6~145.3)	1.3~4.8	2.3~8.3
	20	18.75 (13.96~27.64)	93.73 (69.8~138.1)	1.2~10.1	2.1~12.1
ENNB1	0.2	0.21 (0.15~0.31)	104.08 (72.7~154.9)	1.1~5.9	3.8~10.2
	2	1.76 (1.32~3.14)	88.23 (66.2~156.8)	1.7~9.1	3.6~10.2
	20	17.64 (12.94~26.66)	88.23 (64.7~133.3)	1.4~8.4	4.3~11.3
ENNB	0.2	0.20 (0.14~0.31)	101.13 (71.9~153.4)	2.4~5.6	4.2~10.8
	2	1.81 (1.32~2.53)	90.26 (65.9~126.5)	1.3~10.3	4.3~11.2
	20	19.31 (12.16~35.19)	96.54 (60.8~175.9)	1.9~9.1	2.3~11.5

^1^ R_M_, method recovery.

**Table 5:** Occurrence of emerging mycotoxins in food samples in the 6^th^ China total diet study.

Food		AME	TeA	TEN	AOH	ALT	BEA	ENNA	ENNA1	ENNB	ENNB1
Cereals and cereal products	Samples (n)	5	5	5	5	5	5	5	5	5	5
	Positive (n)	4	5	4	0	0	4	3	3	5	4
	Range (μg kg^−1^)	0.14–10.92	2.60–15.11	0.22–2.28	-	-	0.55–1.95	0.12–0.83	0.10–0.22	0.37–1.05	0.10–0.35
Legume and related products	Samples (n)	5	5	5	5	5	5	5	5	5	5
	Positive (n)	1	3	3	0	0	4	2	2	4	2
	Range (μg kg^−1^)	1.77	0.33–2.79	0.10–0.28	-	-	0.47–5.46	1.03–1.26	0.26–0.29	0.16–0.37	0.36
Potatoes and potato products	Samples (n)	5	5	5	5	5	5	5	5	5	5
	Positive (n)	4	2	1	0	2	2	5	1	4	4
	Range (μg kg^−1^)	0.13–2.40	0.20–0.41	0.96	-	1.08–1.72	2.23–2.58	0.13–1.01	0.15	0.21–0.87	0.11–0.34
Meats and meat products	Samples (n)	5	5	5	5	5	5	5	5	5	5
	Positive (n)	0	0	0	0	0	0	0	0	2	0
	Range (μg kg^−1^)	-	-	-	-	-	-	-	-	0.13–0.24	-
Eggs and egg products	Samples (n)	5	5	5	5	5	5	5	5	5	5
	Positive (n)	2	4	2	0	0	3	3	2	4	4
	Range (μg kg^−1^)	0.72–1.31	0.26–27.73	0.11–27.53	-	-	1.28–6.70	0.20–1.55	0.30–0.51	0.15–0.61	0.18–0.50
Aquatic foods and aquatic food products	Samples (n)	5	5	5	5	5	5	5	5	5	5
	Positive (n)	1	1	2	0	1	2	0	0	3	1
	Range (μg kg^−1^)	0.38	0.68	0.10–0.68	-	8.77	0.32–1.02	-	-	0.29–0.65	0.22
Milk and dairy products	Samples (n)	5	5	5	5	5	5	5	5	5	5
	Positive (n)	0	3	0	0	0	1	0	0	1	0
	Range (μg kg^−1^)	-	1.93–3.00	-	-	-	0.16	-	-	0.26	-
Vegetables and vegetable products	Samples (n)	5	5	5	5	5	5	5	5	5	5
	Positive (n)	2	3	2	0	1	2	0	0	3	2
	Range (μg kg^−1^)	1.10–3.35	0.19–0.71	0.21–0.25	-	1.22	1.85–3.29	-	-	0.20–0.47	0.22–0.26
Fruits and fruit products	Samples (n)	5	5	5	5	5	5	5	5	5	5
	Positive (n)	1	1	0	0	0	1	0	0	0	0
	Range (μg kg^−1^)	0.2	6.62	-	-	-	0.21	-	-	-	-
Sugar and sugar products	Samples (n)	5	5	5	5	5	5	5	5	5	5
	Positive (n)	1	0	0	0	0	0	0	0	0	0
	Range (μg kg^−1^)	0.12	-	-	-	-	-	-	-	-	-
Beverages and water	Samples (n)	5	5	5	5	5	5	5	5	5	5
	Positive (n)	1	0	0	0	0	0	0	0	0	0
	Range (μg kg^−1^)	0.16	-	-	-	-	-	-	-	-	-
Alcohol beverages	Samples (n)	5	5	5	5	5	5	5	5	5	5
	Positive (n)	2	4	3	0	0	1	0	0	0	0
	Range (μg kg^−1^)	0.27–1.00	2.26–17.62	0.14–0.16	-	-	0.12	-	-	-	-

## Supplementary Materials

The following are available online at https://www.mdpi.com/2072-6651/11/3/166/s1, Table S1: Sensitivity, extraction recovery, and matrix effect of the method for each food category. Table S2: Accuracy and precision of the method for each food category.
