# Supplementary material for: Development of Sensitive and Reliable UPLC-MS/MS Methods for Food Analysis of Emerging Mycotoxins in China Total Diet Study
Source: Toxins (Basel). 2019 Mar 17;11(3):166. doi: 10.3390/toxins11030166 (PMC6468665; doi:10.3390/toxins11030166)
Supplement: Supplementary file 1 [file toxins-11-00166-s001.pdf]

Supplementary Materials: Development of Sensitive and Reliable UPLC-MS/MS Methods for Food Analysis of Emerging Mycotoxins in China Total Diet Study

Danlei Sun, Nannan Qiu, Shuang Zhou, Bing Lyu, Shuo Zhang, Jingguang Li, Yunfeng Zhao and Yongning Wu

Table S1. Sensitivity, extraction recovery and matrix effect of the method for each food category.

Analyte	Creals and cereal products					Legume and related products				
	R_E [1] (%)	Matrix Effect (%)	R_A [2] (%)	LOQ ($\mu g\ kg^{-1}$)	LOD ($\mu g\ kg^{-1}$)	R_E [1] (%)	Matrix Effect (%)	R_A [2] (%)	LOQ ($\mu g\ kg^{-1}$)	LOD ($\mu g\ kg^{-1}$)
AME	68.0	117.0	79.6	0.05	0.02	78.2	71.4	55.8	0.08	0.03
TeA	78.8	122.0	96.1	0.1	0.04	89.4	90.7	81.1	0.4	0.1
TEN	95.3	99.7	95.0	0.05	0.02	78.2	121.4	94.9	0.2	0.05
ALT	85.6	105.1	90.1	0.3	0.1	76.3	101.3	77.3	0.3	0.1
AOH	53.7	108.3	58.2	0.5	0.1	69.7	73.9	51.5	0.9	0.3
BEA	68.8	127.3	87.6	0.01	0.003	65.9	162.8	107.3	0.05	0.02
ENNA1	69.6	188.2	131.6	0.007	0.002	63.0	144.1	91.4	0.02	0.007
ENNA	63.4	127.1	80.6	0.007	0.002	65.9	145.1	95.6	0.02	0.004
ENNB1	89.0	156.2	139.0	0.007	0.002	57.8	177.8	102.8	0.02	0.005
ENNB	67.8	164.1	111.3	0.002	0.0004	54.0	166.1	89.7	0.01	0.002

Analyte	Potatoes and potato products					Meats and meat products				
	R_E [1] (%)	Matrix Effect (%)	R_A [2] (%)	LOQ ($\mu g\ kg^{-1}$)	LOD ($\mu g\ kg^{-1}$)	R_E [1] (%)	Matrix Effect (%)	R_A [2] (%)	LOQ ($\mu g\ kg^{-1}$)	LOD ($\mu g\ kg^{-1}$)
AME	73.8	91.0	67.2	0.05	0.01	66.2	128.7	85.2	0.08	0.03
TeA	75.6	86.2	65.2	0.5	0.1	86.2	105.8	91.2	0.9	0.3
TEN	87.7	160.6	140.9	0.06	0.02	89.2	121.3	108.2	0.2	0.05
ALT	90.4	147.8	133.6	0.2	0.04	92.3	149.2	137.7	0.3	0.1
AOH	78.7	103.0	81.1	0.9	0.3	75.1	112.2	84.3	0.9	0.3
BEA	72.2	128.2	92.6	0.04	0.01	66.5	158.9	105.7	0.06	0.02
ENNA1	69.7	184.4	128.5	0.02	0.005	68.3	148.2	101.2	0.06	0.03
ENNA	70.3	153.3	107.8	0.02	0.005	60.7	132.5	80.4	0.06	0.02
ENNB1	64.2	180.9	116.1	0.02	0.004	72.3	166.3	120.2	0.06	0.02
ENNB	72.8	137.7	100.2	0.006	0.002	72.2	134.5	97.1	0.02	0.005

Table S1.*Cont.*

**Analyte**	**Eggs and egg products**					**Aquatic foods and aquatic food products**				
	**R$_E$ [1] (%)**	**Matrix Effect (%)**	**R$_A$ [2] (%)**	**LOQ (µg kg^{-1})**	**LOD (µg kg^{-1})**	**R$_E$ [1] (%)**	**Matrix Effect (%)**	**R$_A$ [2] (%)**	**LOQ (µg kg^{-1})**	**LOD (µg kg^{-1})**
AME	75.2	134.0	100.8	0.04	0.01	68.2	119.8	81.7	0.02	0.005
TeA	78.2	111.3	87.0	0.3	0.1	80.5	93.8	75.5	0.8	0.2
TEN	95.5	107.5	102.7	0.1	0.03	89.9	114.0	102.5	0.07	0.02
ALT	120.7	115.9	139.9	0.2	0.05	84.7	133.1	112.7	0.3	0.1
AOH	65.7	143.5	94.3	0.9	0.3	107.5	103.5	111.3	0.9	0.3
BEA	113.3	134.4	152.3	0.05	0.02	101.9	131.2	133.7	0.02	0.006
ENNA1	110.7	132.4	146.6	0.03	0.01	98.3	122.5	120.4	0.02	0.007
ENNA	117	155.6	182.1	0.02	0.004	100.2	134.2	134.5	0.02	0.005
ENNB1	82.6	149.8	123.7	0.03	0.01	69.2	157.7	109.1	0.03	0.01
ENNB	67.2	131.1	88.1	0.006	0.002	64.9	151.9	98.6	0.04	0.01
**Analyte**	**Milk and dairy products**					**Vegetables and vegetable products**				
	**R$_E$ [1] (%)**	**Matrix Effect (%)**	**R$_A$ [2] (%)**	**LOQ (µg kg^{-1})**	**LOD (µg kg^{-1})**	**R$_E$ [1] (%)**	**Matrix Effect (%)**	**R$_A$ [2] (%)**	**LOQ (µg kg^{-1})**	**LOD (µg kg^{-1})**
AME	62.4	122.4	76.4	0.08	0.03	75.5	97.4	73.5	0.04	0.01
TeA	82.3	119.8	98.6	0.4	0.1	86.9	86.9	75.5	0.5	0.2
TEN	99.5	108.5	108.0	0.2	0.05	81.4	162.7	132.4	0.1	0.03
ALT	86.6	182.4	158.0	0.3	0.08	73.2	158.9	116.3	0.5	0.2
AOH	82.5	104.3	86.0	0.9	0.3	83.1	98.8	82.1	0.6	0.2
BEA	70.0	141.8	99.3	0.05	0.02	67.8	86.7	58.8	0.03	0.008
ENNA1	66.2	122.6	81.2	0.02	0.007	89.3	71.4	63.8	0.04	0.1
ENNA	61.7	138.6	85.5	0.02	0.004	80.4	72.3	58.1	0.03	0.007
ENNB1	65.3	196.7	128.4	0.02	0.005	65.4	127.6	83.5	0.05	0.02
ENNB	70.4	167.3	117.8	0.008	0.002	63.4	167.0	105.9	0.01	0.004

Table S1. *Cont.*

Analyte	Fruits and fruit products					Sugar and sugar products				
	R_E [1] (%)	Matrix Effect (%)	R_A [2] (%)	LOQ (μg kg^{-1})	LOD (μg kg^{-1})	R_E [1] (%)	Matrix Effect (%)	R_A [2] (%)	LOQ (μg kg^{-1})	LOD (μg kg^{-1})
AME	68.4	122.8	84.0	0.02	0.006	64.6	108.7	70.2	0.05	0.02
TeA	80.4	92.1	74.0	0.2	0.06	82.1	86.9	71.3	0.2	0.05
TEN	87.9	123.3	108.4	0.2	0.04	82.6	114.0	94.2	0.1	0.03
ALT	76.0	190.7	144.4	0.9	0.3	85.8	98.5	84.5	0.2	0.06
AOH	67.1	123.4	82.8	0.8	0.2	74.3	112.7	83.7	0.9	0.3
BEA	64.5	98.3	63.4	0.06	0.02	51.3	174.8	89.7	0.02	0.004
ENNA1	64.0	141.4	90.5	0.02	0.005	55.5	145.2	80.6	0.02	0.006
ENNA	69.0	129.5	89.4	0.009	0.003	54.4	121.9	66.4	0.01	0.003
ENNB1	87.8	123.3	108.3	0.04	0.01	56.0	193.7	108.5	0.02	0.006
ENNB	110.7	122.0	135.1	0.02	0.005	59.3	170.0	100.8	0.02	0.004
Analyte	Beverages and water					Alcohol beverages				
	R_E [1] (%)	Matrix Effect (%)	R_A [2] (%)	LOQ (μg kg^{-1})	LOD (μg kg^{-1})	R_E [1] (%)	Matrix Effect (%)	R_A [2] (%)	LOQ (μg kg^{-1})	LOD (μg kg^{-1})
AME	75.5	98.1	74.1	0.01	0.003	70.6	113.0	79.8	0.01	0.003
TeA	85.8	71.8	61.6	0.6	0.2	100.8	65.6	66.2	0.2	0.04
TEN	84.5	156.6	132.3	0.1	0.04	93.6	142.6	133.5	0.06	0.02
ALT	82.2	183.7	151.0	0.2	0.05	102.5	140.8	144.3	0.3	0.1
AOH	69.0	86.2	59.5	0.4	0.1	64.4	109.1	70.3	0.9	0.3
BEA	71.5	74.5	53.3	0.08	0.002	78.6	125.7	98.8	0.06	0.02
ENNA1	74.3	86.4	64.2	0.02	0.006	63.0	137.9	86.9	0.02	0.004
ENNA	64.1	75.5	48.4	0.01	0.003	71.2	126.4	90.0	0.008	0.003
ENNB1	66.9	114.4	76.5	0.01	0.003	69.8	115.5	80.6	0.02	0.004
ENNB	64.3	115.7	74.4	0.01	0.003	72.2	155.5	112.3	0.003	0.001

[1] R_E, extraction recovery; [2] R_A, apparent recovery.

Table S2. Accuracy and precision of the method for each food category.

Analyte	Cereals and cereal products					Legume and related products			
	Spiked level (ng/mL)	Measured value (ng/mL)	R_M [1] (%)	RSD (%)		Measured value (ng/mL)	R_M [1] (%)	RSD (%)	
				Intra-day	Inter-day			Intra-day	Inter-day
AME	2	1.26	62.8	2.9	4.2	1.66	83.0	11.8	12.2
	20	14.47	72.3	1.2	5.2	14.79	74.0	4.8	6.6
	200	188	94.0	2.4	5.2	131.0	65.5	7.2	8.2
TeA	2	2.45	122.6	3.4	5.6	1.93	96.3	4.3	6.5
	20	18.38	91.9	1.5	3.3	18.60	93.0	1.5	3.2
	200	180.6	90.3	0.9	3.2	176.5	88.2	5.2	6.6
TEN	2	2.49	124.6	5.9	10.8	2.02	101.1	9.5	11.8
	20	20.48	102.4	2.0	5.3	19.18	95.9	2.9	5.6
	200	184.8	92.4	1.9	3.4	168.8	84.4	1.9	2.3
ALT	2	2.25	112.4	2.4	5.6	2.28	114.0	1.6	5.8
	20	25.26	126.3	3.8	6.3	17.26	86.3	1.6	6.6
	200	257.2	128.6	4.2	6.8	156.6	78.3	6.5	7.8
AOH	2	1.77	88.5	5.2	11.2	2.78	139.1	4.1	6.2
	20	14.72	73.6	8.5	10.9	13.65	68.3	11.1	12.3
	200	20.02	100.1	1.6	4.2	151.8	75.9	4.7	6.8
BEA	0.2	0.3	149.1	2.8	4.8	0.25	123.9	9.6	11.2
	2	1.96	97.8	2.5	4.1	1.37	68.4	7.7	9.6
	20	14.07	70.4	3.6	6.2	12.31	61.5	5.6	6.2
ENNA1	0.2	0.31	154.3	3.3	6.3	0.168	84.0	8.1	9.8
	2	2.05	102.4	2.6	7.8	1.405	70.2	3.5	3.9
	20	20.86	104.3	2.3	5.6	12.24	61.2	4.6	5.6
ENNA	0.2	0.33	164.4	6.5	8.8	0.13	64.3	5.4	9.5
	2	2.57	128.6	4.5	5.2	1.23	61.6	2.7	6.4
	20	18.72	93.6	10.1	12.0	13.96	69.8	9.9	12.1
ENNB1	0.2	0.24	121.9	4.2	8.8	0.15	72.7	1.1	3.8
	2	2.20	110.0	9.1	10.2	1.55	77.5	1.7	3.6
	20	20.36	101.8	7.4	10.6	12.94	64.7	3.2	5.6
ENNB	0.2	0.28	138.8	2.6	4.2	0.15	72.8	4.2	9.5
	2	2.43	121.5	3.5	8.3	1.48	73.9	7.5	7.9

	20	25.37	126.9	5.8	8.5	12.16	60.8	1.9	2.3

Table S2. *Cont.*

Analyte	Potatoes and potato products					Meats and meat products			
	Spiked level (ng/mL)	Measured value (ng/mL)	R_M [1] (%)	RSD (%)		Measured value (ng/mL)	R_M [1] (%)	RSD (%)	
				Intra-day	Inter-day			Intra-day	Inter-day
AME	2	1.46	72.9	3.8	4.6	1.53	76.5	2.2	8.2
	20	15.25	76.3	5.2	8.7	15.84	79.2	1.5	5.4
	200	167.4	83.7	4.5	9.5	164.6	82.3	7.3	9.2
TeA	2	1.62	81.0	1.5	4.2	2.16	108.2	1.5	6.8
	20	19.69	98.5	8.2	8.6	20.25	101.3	4.6	8.3
	200	178.4	89.2	5.5	6.8	194.0	97.0	2.9	5.4
TEN	2	2.20	109.8	2.3	5.6	2.26	113.1	4.6	8.2
	20	23.39	117.0	1.2	3.3	22.58	112.9	3.5	10.8
	200	167.3	83.7	1.6	4.8	216.5	108.3	1.8	2.3
ALT	2	2.68	134.0	1.8	3.6	2.34	117.1	3.8	7.2
	20	25.25	126.2	6.6	8.8	22.68	113.4	4.6	8.2
	200	215.8	107.9	4.2	7.2	161.2	80.6	3.6	4.8
AOH	2	2.39	119.3	8.1	10.3	1.44	72.3	4.5	8.6
	20	19.1	95.5	4.2	5.8	17.62	88.1	7.2	9.5
	200	225.9	112.9	9.8	10.2	166.0	83.0	1.5	6.9
BEA	0.2	0.17	84.4	7.5	8.6	0.28	141.2	1.5	6.8
	2	1.99	99.3	2.5	5.5	2.64	132.2	7.2	9.5
	20	16.77	83.9	3.2	5.3	22.35	111.8	4.3	8.2
ENNA1	0.2	0.3	150.3	4.5	7.8	0.25	125.6	1.5	5.5
	2	2.08	104.2	5.5	8.2	2.57	128.5	2.2	4.3
	20	15.16	75.8	7.4	10.3	26.81	134.1	9.2	11.5
ENNA	0.2	0.22	107.9	2.6	5.3	0.28	141.5	6.3	4.8
	2	2.66	133.1	2.8	4.8	2.39	145.3	3.2	4.3
	20	14.08	70.4	3.8	2.1	27.64	138.1	1.2	5.8
ENNB1	0.2	0.31	154.9	3.2	5.2	0.16	79.2	4.5	6.4
	2	3.14	156.8	5.8	6.6	1.72	86.9	4.5	8.8
	20	16.37	81.9	6.8	6.9	23.66	118.3	6.2	10.6
ENNB	0.2	0.27	136.7	5.2	10.5	0.25	125.3	3.8	4.5

	2	2.03	101.7	1.3	5.6	2.53	126.5	2.8	6.3
	20	16.2	81.0	2.6	5.8	32.19	161.0	7.7	11.2

Table S2. *Cont.*

Analyte	Eggs and egg products					Aquatic foods and aquatic food products			
	Spiked level (ng/mL)	Measured value (ng/mL)	R_M [1] (%)	RSD (%)		Measured value (ng/mL)	R_M [1] (%)	RSD (%)	
				Intra-day	Inter-day			Intra-day	Inter-day
AME	2	1.54	76.9	5.8	10.5	1.63	81.4	1.2	5.5
	20	15.06	75.3	3.8	4.6	15.24	76.2	2.2	5.6
	200	166.3	83.1	2.8	5.2	154.6	77.3	6.5	7.7
TeA	2	2.58	129.1	4.2	6.3	2.06	102.8	3.2	5.5
	20	17.38	86.9	3.8	9.5	20.05	100.3	2.8	8.4
	200	179.9	89.9	2.4	6.4	184.0	92.0	2.5	6.2
TEN	2	2.30	115.1	2.8	4.4	2.40	120.1	3.8	4.9
	20	25.32	126.6	3.5	8.2	22.88	103.4	3.9	7.2
	200	229.8	114.9	3.6	7.8	210.9	75.8	5.3	6.5
ALT	2	1.71	85.5	5.2	8.5	2.44	122.1	3.4	7.9
	20	22.78	113.9	2.4	9.9	20.68	103.4	7.8	9.3
	200	194.5	97.2	7.5	9.3	151.5	75.8	7.8	10.2
AOH	2	1.41	70.7	4.5	7.2	2.88	143.8	3.4	6.4
	20	13.59	67.9	6.2	10.2	17.12	85.6	3.5	7.4
	200	136.2	68.1	1.6	9.2	162.8	81.4	3.6	7.2
BEA	0.2	0.27	134.8	7.3	9.9	0.23	113.7	2.8	10.8
	2	2.2	110.2	2.5	8.5	2.04	102.2	5.6	9.5
	20	23.94	119.7	5.6	8.8	24.75	123.8	2.9	10.9
ENNA1	0.2	0.25	127.2	6.8	9.2	0.21	102.6	7.2	6.3
	2	2.11	105.3	1.5	8.3	1.57	78.5	4.8	8.5
	20	20.14	100.7	5.2	8.4	25.41	127.0	3.6	6.4
ENNA	0.2	0.25	124.8	3.4	8.2	0.18	90.0	5.2	8.4
	2	2.06	102.9	3.6	6.3	1.99	99.4	3.8	6.2
	20	21.02	105.1	7.2	10.3	26.44	132.2	1.2	7.9
ENNB1	0.2	0.3	147.6	4.9	8.5	0.16	78.8	2.5	4.3
	2	1.32	66.2	3.8	8.3	1.42	70.9	2.1	4.9
	20	15.41	77.1	2.8	5.3	26.66	133.3	3.8	9.1

ENNB	0.2	0.31	153.4	4.8	6.6	0.23	116.0	2.4	6.1
	2	1.88	94	2.3	9.2	2.35	117.4	8.2	9.6
	20	12.67	63.3	9.1	10.4	35.19	175.9	9.1	11.5

Table S2. *Cont.*

Analyte	Milk and dairy products					Vegetables and vegetable products			
	Spiked level (ng/mL)	Measured value (ng/mL)	R_M [1] (%)	RSD (%)		Measured value (ng/mL)	R_M [1] (%)	RSD (%)	
				Intra-day	Inter-day			Intra-day	Inter-day
AME	2	2.63	131.1	5.2	6.3	1.41	70.5	5.2	9.5
	20	21.86	109.3	5.1	8.6	14.36	71.8	2.5	6.6
	200	166.6	83.3	2.4	4.2	140.9	70.5	3.8	4.2
TeA	2	1.91	95.4	1.8	6.3	1.88	94.2	6.9	9.3
	20	18.63	93.2	3.5	5.9	17.5	87.5	3.2	5.5
	200	180.1	90.0	6.4	10.1	171.4	85.7	3.4	6.4
TEN	2	2.46	123.2	3.8	7.4	2.26	112.8	7.2	9.5
	20	21.50	107.5	5.2	10.2	20.06	100.3	2.8	3.2
	200	213.1	106.6	3.7	5.8	205.8	102.9	6.3	9.8
ALT	2	1.83	91.5	2.6	8.5	2.69	134.6	8.1	10.2
	20	15.98	80.0	6.4	9.2	24.00	120.0	1.2	6.9
	200	218.5	109.3	3.5	6.3	199.5	99.8	3.5	4.3
AOH	2	2.56	127.9	8.2	9.1	1.62	81.0	2.1	6.2
	20	19.57	97.8	6.5	8.2	24.16	120.8	3.5	6.4
	200	14.95	74.7	2.3	7.2	25.76	128.8	6.1	9.2
BEA	0.2	0.22	112.4	2.6	6.9	0.15	75.3	7.2	10.5
	2	2.84	141.9	3.5	7.6	1.99	99.7	2.4	7.1
	20	15.15	75.7	2.6	9.5	14.72	73.6	6.2	8.8
ENNA1	0.2	0.26	127.6	4.5	4.2	0.26	128.5	1.2	3.7
	2	2.02	100.8	1.3	6.3	1.83	91.4	2.4	2.5
	20	21.59	107.9	4.2	9.8	14.11	70.5	5.1	8.4
ENNA	0.2	0.26	130.5	3.4	7.2	0.15	75.5	3.2	10.1
	2	2.70	135.1	3.2	6.3	1.51	75.5	2.5	7.1
	20	26.02	130.1	8.2	11.3	14.23	71.2	7.2	11.2
ENNB1	0.2	0.24	117.8	5.9	8.1	0.17	87.1	4.2	10.2
	2	1.97	98.4	2.4	6.3	1.95	97.4	3.1	5.8

	20	21.85	109.3	2.1	4.3	14.32	71.6	2.4	4.6
ENNB	0.2	0.19	93.9	5.2	7.6	0.14	71.9	4.1	6.6
	2	1.56	77.9	3.5	7.2	1.36	68.2	2.1	10.5
	20	18.01	90.0	5.8	11.4	16.73	83.7	5.8	6.3

Table S2. *Cont.*

Analyte	Fruits and fruit products					Sugar and sugar products			
	Spiked level (ng/mL)	Measured value (ng/mL)	R_M [1] (%)	RSD (%)		Measured value (ng/mL)	R_M [1] (%)	RSD (%)	
				Intra-day	Inter-day			Intra-day	Inter-day
AME	2	2.28	114.0	3.5	5.5	2.01	100.7	1.5	3.6
	20	14.69	73.3	2.4	3.6	19.19	95.9	3.2	8.6
	200	144.9	72.4	2.1	2.8	176.0	88.0	2.8	5.2
TeA	2	1.98	98.9	3.3	5.3	1.92	96.0	3.4	9.3
	20	17.07	85.3	8.2	10.9	18.89	94.5	1.8	5.5
	200	173.2	86.6	6.1	7.6	179.8	89.9	6.4	7.8
TEN	2	2.41	120.6	2.5	6.4	1.87	93.5	3.2	4.6
	20	21.19	105.9	9.2	10.8	16.35	81.8	2.1	6.4
	200	209.0	104.5	3.5	9.8	176.8	88.4	3.1	7.1
ALT	2	2.17	108.7	2.4	10.5	2.54	126.9	2.8	6.5
	20	22.42	112.1	3.9	9.7	25.42	127.1	3.2	7.6
	200	252.4	126.2	2.1	4.4	232.4	116.2	2.4	6.9
AOH	2	1.65	82.4	5.4	7.2	1.97	98.7	4.2	9.1
	20	23.33	116.7	3.6	8.1	15.29	76.5	2.4	8.2
	200	20.66	103.3	2.7	8.2	162.7	81.4	1.5	5.2
BEA	0.2	0.25	123.0	3.9	7.5	0.26	133.3	1.7	6.3
	2	1.31	65.3	2.6	7.9	1.54	76.8	3.8	9.2
	20	13.90	69.5	9.1	10.3	15.70	78.5	7.5	10.8
ENNA1	0.2	0.25	123.5	2.1	2.4	0.19	94.2	6.2	8.8
	2	1.33	66.6	1.6	6.3	1.66	82.9	2.5	5.1
	20	14.92	74.6	5.1	4.4	16.88	84.4	5.1	7.3
ENNA	0.2	0.29	144.5	3.9	5.6	0.21	105.9	3.2	9.7
	2	1.54	77.1	3.7	8.3	1.60	79.9	4.8	2.3
	20	15.52	77.6	3.9	8.3	16.85	84.2	8.7	11.8
ENNB1	0.2	0.23	114.9	5.2	9.4	0.21	105.3	2.3	9.5

	2	1.34	66.9	2.5	3.8	1.62	80.9	5.7	9.1
	20	14.12	70.6	2.1	6.3	14.00	70.0	3.6	5.1
ENNB	0.2	0.15	77.2	4.2	7.6	0.16	82.2	5.6	10.8
	2	1.32	65.9	5.1	6.2	1.60	80.0	2.1	4.3
	20	13.96	69.8	5.6	7.4	16.59	82.9	6.6	11.4

Table S2. *Cont.*

Analyte	Beverages and water					Alcohol beverages			
	Spiked level (ng/mL)	Measured value (ng/mL)	R_M [1] (%)	RSD (%)		Measured value (ng/mL)	R_M [1] (%)	RSD (%)	
				Intra-day	Inter-day			Intra-day	Inter-day
	2	1.46	73.2	3.1	5.4	1.31	65.5	4.8	6.5
AME	20	15.58	77.9	2.2	6.2	16.61	83.0	3.2	5.8
	200	149.8	74.9	7.6	10.3	157.0	78.5	6.2	8.2
	2	1.77	88.46	2.4	6.4	2.53	126.4	1.2	3.3
TeA	20	18.65	93.2	1.4	4.6	18.52	92.6	3.1	4.5
	200	178.5	89.3	7.8	10.5	172.6	86.3	2.7	6.4
	2	2.06	103.1	2.3	5.5	2.52	125.9	2.2	5.4
TEN	20	19.98	99.9	3.0	6.3	26.34	131.7	1.6	3.2
	200	222.8	114.2	4.4	5.8	232.9	116.4	2.6	8.8
	2	1.99	99.7	5.3	10.2	2.41	120.5	3.7	4.5
ALT	20	17.45	87.2	8.4	11.3	24.31	121.6	4.5	6.9
	200	222.6	111.3	1.6	5.3	199.4	99.7	5.7	7.3
	2	1.57	78.6	2.2	4.8	2.67	133.4	4.8	6.2
AOH	20	14.1	70.5	2.5	5.1	14.08	70.4	6.0	10.2
	200	136.7	68.3	3.5	6.2	166.4	83.2	1.5	3.2
	0.2	0.15	75.3	2.8	5.8	0.13	63.1	7.4	8.9
BEA	2	1.55	77.3	4.3	7.5	1.35	67.6	2.0	4.5
	20	14.94	74.7	2.1	5.9	12.69	63.5	6.5	8.8
	0.2	0.18	89.7	1.8	4.5	0.13	64.5	8.1	10.2
ENNA1	2	1.49	74.6	5.6	8.8	1.21	60.6	1.3	3.3
	20	17.15	85.8	2.4	6.9	12.22	61.1	2.5	5.4
	0.2	0.17	82.5	3.8	8.6	0.12	60.3	3.3	6.2
ENNA	2	1.54	77.2	1.3	3.5	1.3	64.8	2.7	5.3
	20	16.47	82.3	2.3	6.2	14.0	70.2	7.5	10.3

	0.2	0.17	84.6	1.4	5.5	0.17	84.2	3.9	5.5
ENNB1	2	1.42	70.8	2.9	5.6	1.52	76.0	4.5	8.2
	20	16.91	84.6	1.4	8.3	15.1	75.5	8.4	11.3
	0.2	0.15	72.9	3.2	5.9	0.14	72.5	3.8	5.6
ENNB	2	1.35	67.3	5.5	10.2	1.77	88.8	10.3	11.2
	20	16.77	83.9	7.3	11.2	15.87	79.3	2.1	5.4

[1] R_M, method recovery.
